# Renal Cell Carcinoma Arising from Isthmus of Horseshoe K

**DOI:** 10.15586/jkcvhl.v10i2.267

**Published:** 2023-05-02

**Authors:** Sanjay M. Khaladkar, Sai Sabari Vinay Kumar Parripati, Deepak Koganti, Satvik Dhirawani, Urvashi Agarwal

**Affiliations:** Department of Radio-Diagnosis, Dr. D. Y. Patil Medical College, Hospital & Research Centre, Dr. D. Y. Patil Vidyapeeth, Pune, India

**Keywords:** fusion anomaly, horseshoe kidney, renal cell carcinoma

## Abstract

The most common congenital renal fusion anomaly is the horseshoe kidney (HSK) occurring in about 1 in 600–700 individuals in the Indian population. HSKs are associated with problems such as renal stones, obstruction of uretero-pelvic junction causing stasis, and infection due to ectopic location of the kidneys, malrotation of the kidneys, and vascular changes. In general, normally developed kidneys have more incidents of renal cell carcinoma (RCC) as compared to HSKs. The major issue arises during surgery of HSK due to their altered anatomy and aberrant blood supply. We present a case of HSK with RCC located in the isthmus of a 43-year-old woman.

## Introduction

The most frequent renal fusion abnormality is the horseshoe kidney (HSK) (1). Ectopic location of kidneys, malrotation, and vascular changes are the three anatomical abnormalities that are combined to form the HSK (2). Infections, stasis, ureteropelvic junction blockage, and renal calculi are all conditions that are more common in HSKs than in normal ones. Males are more likely to develop a HSK than females (3). Patients with fusion abnormalities have three-to-four-folds increased chance of developing nephroblastoma and urothelial tumors. In the HSK, clear cell carcinoma makes up the majority of the renal cell tumors (4). However, in our case, the renal cell carcinoma (RCC) is located in the isthmus of the HSK and is of papillary cell subtype, which makes the case unique.

## Case Presentation

**Figure 1:**
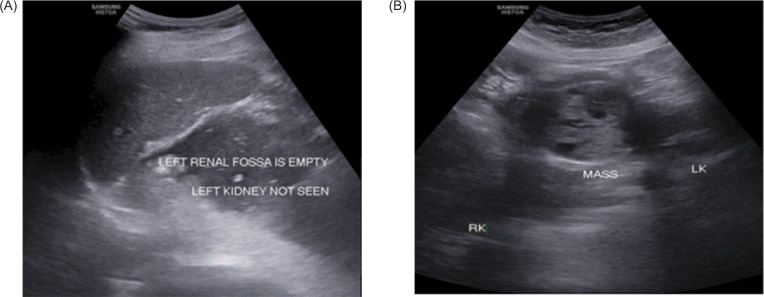
(A) Left kidney not visualized in the left renal fossa; (B) Well-defined solid mass of heterogeneous echotexture arising from isthmus of HSK.

A 43-year-old female patient presented to the Medicine Department complaining of right flank colic that has been persisting for 2 months with a sudden onset and of moderate intensity. The patient also gave history of fever with chills, nausea, and episodes of non-bilious vomiting since 7 days. The patient also complained of burning micturition since 1 month. There was no history of hematuria. No known comorbities were noted. Base-line blood investigations and urine routine examinations were normal. Ultrasound examination of the abdomen revealed HSKs, which were slightly unascended with fusion of both the lower poles of the kidneys by normal renal parenchyma connecting across midline anterior to aorta and inferior vena cava (IVC). A well-defined sold mass of heterogeneous echotexture with few hypoechoic areas within the lesion was seen arising from the upper portion of isthmus (Figure 1).

**Figure 2:**
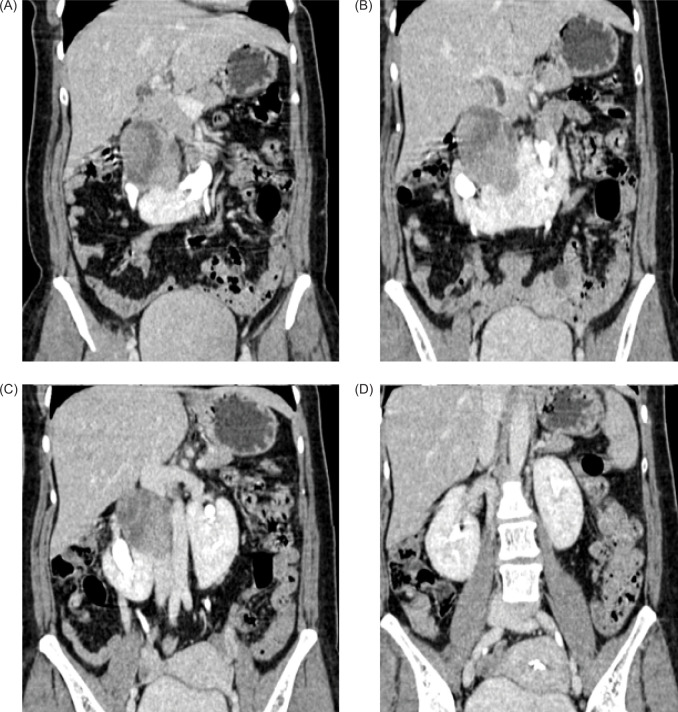
Contrast-enhanced CT coronal sections showing medially deviated lower poles of the bilateral kidneys with heterogenous enhancing mass arising from the superior portion of isthmus of the HSK abutting the hilum of right kidney.

Plain and contrast-enhanced CT scan of abdomen was performed for further evaluation. It revealed HSKs with lower pole of both kidneys deviated medially and fused in midline with renal parenchyma measuring 2.1 cm in thickness and 3.3 cm in cranio-caudal extent. Calyces of lower pole of both kidneys were directed medially. Left kidney showed a bifid renal pelvis. A large, well-defined solid mass of heterogeneous density showing heterogeneous post-contrast enhancement measuring 66 x 54 x 57 mm (transverse x anteroposterior x craniocaudal, respectively) was noted arising from the superior portion of isthmus and extending superiorly at adjoining retroperitoneum and abutting medial surface of right kidney up to renal hilum with obscuration of intervening fat plane. It was extending anterior to right renal hilum and abutting anterosuperior surface of right renal pelvis (Figure 2).

**Figure 3:**
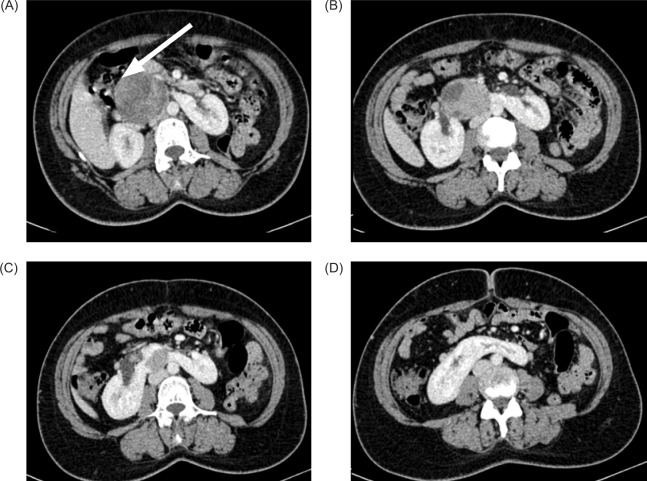
(A–D) Axial contrast enhanced CT abdomen showing well-defined heterogeneously enhancing solid mass arising from isthmus compressing and displacing adjoining second and proximal third portion of the duodenum (arrow in [A]).

This mass was causing extrinsic compression and anterior displacement of distal portion of second part and proximal portion of third part of duodenum that were compressed and displaced anteriorly (Figure 3).

**Figure 4:**
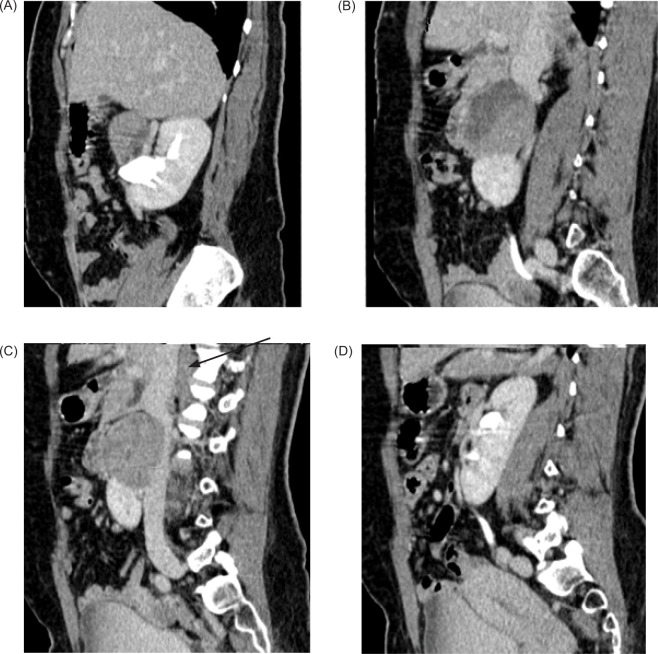
(A–D) Contrast enhanced CT sagittal images—Mass arising from the isthmus, posteriorly compressing the IVC with obliteration of intervening fat planes (arrow).

The pancreatic head was compressed and displaced anteriorly; posteriorly mass effect was noted on anterior surface of inferior vena cava with obliteration of intervening fat plane. IVC as a result was compressed and flattened anteroposteriorly (Figure 4).

On the right side, the mass was extending anterior to the right renal hilum and abutting anterossuperior surface of right renal pelvis.

A diagnosis of HSK with a large solid heterogenous enhancing mass arising from superior portion of isthmus, likely a RCC, was made.

**Figure 5:**
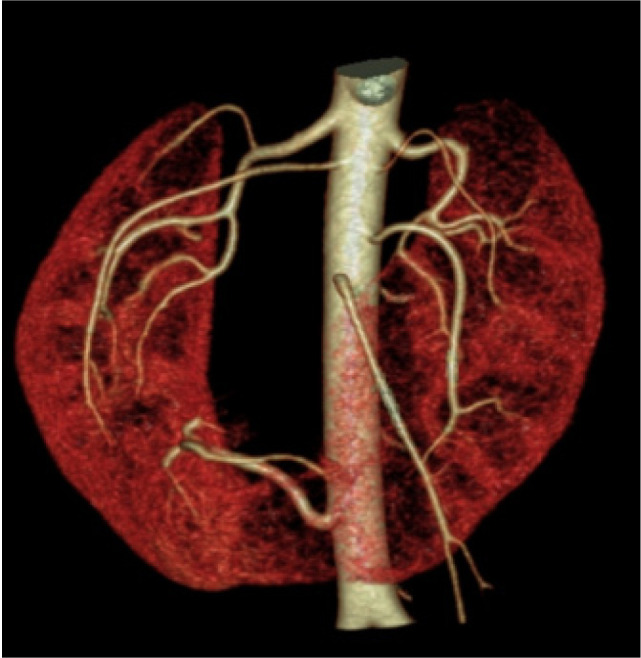
Three-dimensional volume rendering CT angiography showing renal and accessory arteries on either sides supplying both kidneys. Another artery arising from infra renal aorta supplying isthmus, tumor, and adjoining lower pole of right kidney.

Furthermore, the patient was subjected to CT renal angiography which revealed one main renal artery showing hilar branching supplying right upper and interpolar region, and right accessory renal artery supplying interpolar and lower polar region. Left main renal artery showed hilar branching supplying the left interpolar and lower polar region. Out of the two left renal accessory arteries, one artery supplied the left interpolar and lower polar region and the other artery supplied the left upper pole. Another artery arising from infra renal abdominal aorta on the right side at the level of third lumbar vertebra trifurcated the medial branch that supplied isthmus, middle branch that supplied tumor, and lateral branch that supplied the right kidney (Figure 5). Patient could not afford PET-CT, and TNM staging revealed PT1bN0M0 Stage 1.

The patient was subjected to right radical nephrectomy along with isthmectomy. Intraoperatively, the right line of Todd’s incision was taken. Mass was seen in the isthmus extending to adjoining retroperitoneum. Fat planes between mass and great vessels were preserved. Dissection of mass along with some part of the isthmus was perfomed. Complete macroscopic removal of mass done, and closure was done in layers.

Mass specimen was sent for histopathological examination, which revealed papillary RCC type 1, Grade G1, pathological stage pT3a.

## Discussion

HSK is commonly seen in males with male to female ratio of 2:1. Its incidence is 0.2–0.3% of the general population and is a common renal congenital anomaly (5). HSK can be seen in chromosomal disorders such as Down’s syndrome, Edward’s syndrome, and Turner and Patau syndrome (6).

In HSK, two distinct kidneys are connected by normal renal parenchyma or fibrous isthmus across midline. From an embryological perspective, it happens after the ureteric bubs penetrate the renal blastema, which happens between the fourth and sixth weeks of gestation. KolambutBotallo published the first thorough description and depiction of a HSK in 1564, 4 years after Jacopo Berengario da Carpi initially mentioned it during an autopsy in 1522. The kidneys commonly join at their lower poles in 95%, while isthmus connects both upper poles in 5% (7).

Relative risk of malignancy in HSK is increased as compared to non-fused units (8). Malignant tumors seen in HSK include adenocarcinoma, transitional cell carcinoma, Wilms’ tumor, squamous cell carcinoma, lymphoma, and sarcoma. Of these, incidence rate of adenocarcinoma is high. Usually the tumor is seen in one unit of the fused kidney. Occasionally, synchronous bilateral renal cell tumor can occur in HSK (3).

Less than 200 cases of malignancies in HSKs have been documented in English literature (9). Four incidences of tumors in crossing, fused ectopias have been recorded. RCC is the most frequently reported tumor, yet its incidence is less comparable to that of the general population. There is an increased risk of developing nephroblastoma and transitional cell carcinoma in HSK due to stasis, infection, urolithiasis, and embryogenic mechanisms (10).

Schubert et al. published the largest collection of cases involving HSK tumors. Although uncommon, there have been reports of bilateral HSK tumors in the literature. Additionally, there were two examples of HSKs where the tumor was located on the cortical fusion-isthmus (11).

There have been three cases of the HSK tumor documented by Fazio et al. In two patients, histological results for RCC were found, while the histopathological findings of the other revelaed transitional cell carcinoma (12).

In 2015, Kongnyuy et al. described a tumor at the intersection of the inferior pole of the right renal component of the HSK and the renal isthmus (13).

In 2017, Grygorenko et al. reported partial nephrectomy of the HSK with localized RCC in the isthmus and lower poles of both halves (14).

The prognosis of tumor is similar to prognostic factor as in the case of normal kidneys. During incomplete ascent, HSK can receive blood supply from several sources such as aorta, iliac artery, renal artery, and inferior mesenteric artery. The isthmus usually has its own blood supply. It is possible for many aberrant arteries to traverse the pelviureteric junction and the proximal ureter, leading to blockage, urinary stasis, infection, and calculus formation. CT scan and CT angiography are essential in diagnosing tumor in HSK or cross-fused ectopia. Both help in diagnosing uncertain anatomy and knowing the blood supply of kidneys and isthmus. This helps in surgical approach as division of the isthmus is mandatory to gain access to the tumor and surrounding lymph nodes. Vascular supply is difficult to predict during surgery as aberrant vascular supply is common in HSK. Preoperative angiography (Digital subtraction/CT/MR angiography) is mandatory prior to radiologic and surgical intervention for the preoperative assessment as these can detect minor anomalous arteries. This helps in avoiding extensive hemorrhage during surgery (15).

There are six different patterns of arterial supply to HSK. Isthmus usually has dual arterial supply. Each half of the isthmus has a dedicated arterial supply (13).

**Figure 6:**
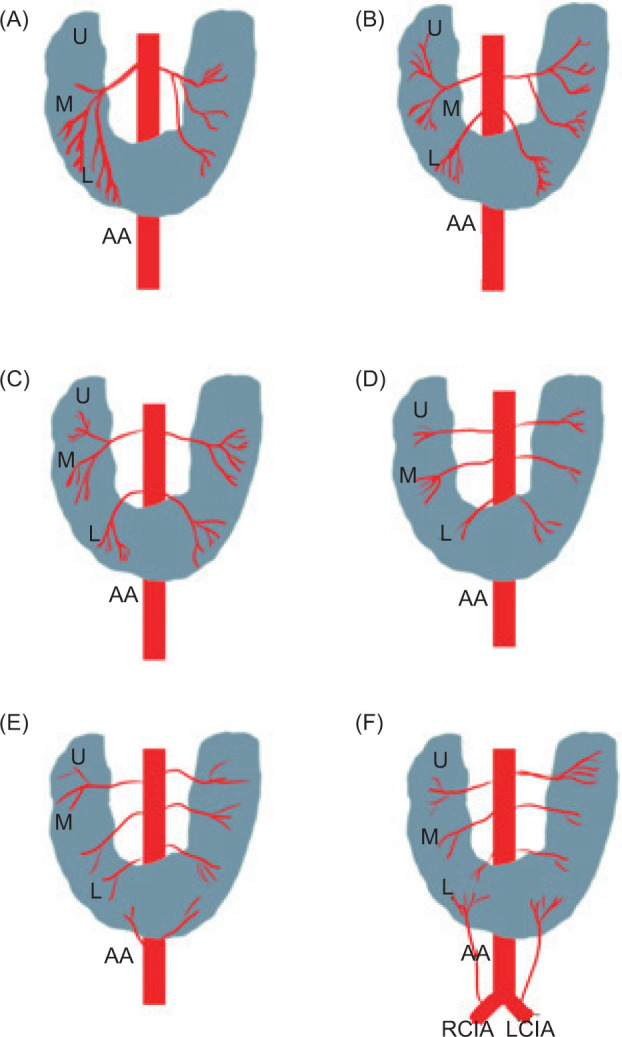
The basic arterial patterns of supply to horseshoe kidney as described by Graves. U: upper, M: middle, L: lower, AA: abdominal aorta, RCIA: right common iliac artery, LCIA: left common iliac artery.

Graves’ classification has six basic patterns of arterial supply for each HSK segment. Each moiety has upper, middle, and lower segments (2, 13). See Figure 6.

Preoperative renal artery embolization can be done to preserve maximum normal renal parenchyma. Limited resection or heminephrectomy can be done in tumors in HSK with special attention to arterial supply and renal pelvis (16). An efficient tool in organ-preserving surgery, preoperative super selective renal artery embolization, aids in the diagnosis of the tumor site due to discoloration, prevents bleeding issues during the procedure, and permits the preservation of the greatest amount of normally functioning renal parenchyma (17).

There is a possibility of tumor extending from one moiety to involve another moiety via isthmus. The isthmus is typically involved in two out of every three cases of tumors in the HSK (4). Surgery of both kidneys along with isthmus is needed in such cases. Hematoma and abscess are frequently associated with each other. The unaffected kidney should not be affected by ischemia. The surgical treatment of choice for the majority of affected people is heminephrectomy, accompanied by isthmus resection and lymphadenectomy (15).

To carry out complete resection of the tumor, preoperative information about tumor localization, its vasculature, and staging (extension in adjoining structures) is mandatory. This helps in avoiding removal of functional tissue. If tumor is confined to isthmus and is of small size, isthmectomy is feasible. Surgery for both renal moieties is needed if the tumor is present on both sides of the isthmus. Whether the surgery is organ sparing or radical, the division of the isthmus is needed to access the lymph nodes, normalize the course of the ureters, and prevent development of Rovsing syndrome. Prognosis depends on the stage of the tumor and its type rather than the renal anomaly. RCC in HSK can be of clear cell, papillary, chromophobe, and oncocytoma varieties, most common being the clear cell type (18). The papillary variety constitutes 13–15% of the RCCs in normal kidneys. Papillary RCC is of two types, type I and type II, with type II being less common than type I (7).

The presence of a HSK can make access to the kidney more difficult during surgery, particularly when using minimally invasive techniques such as laparoscopy or robotic surgery. Surgeons may need to modify their approach to ensure adequate exposure and avoid injury to the surrounding structures (19).

In a retrospective, multicenter cohort study of 43 HSK tumors in 40 patients, Roussel et al. found that enucleation, enucleoresection, and wedge resection can be used to remove the tumor with the greatest amount of functional renal parenchyma preservation, depending on its location and complexity of the tumor. Of the eight patients who underwent minimally invasive surgery (MIS), 32 underwent open surgery. A heminephrectomy with isthmus division is preferred in some circumstances. It is also possible to divide the isthmus using MIS, when the isthmus is divided with stapling tools or by transection and emostatic sutures. Those with more complicated tumors may need an open approach (18).

In patients with HSK and RCC, surgical treatment should aim to preserve as much of the functioning kidney tissues as possible, to minimize the risk of chronic kidney disease and end-stage renal failure. Partial nephrectomy, when feasible, is the preferred surgical approach.

Large tumors that are difficult to remove with open surgery from the intraperitoneal access, or in some situations from the lumbar posterolateral access, should be considered. These factors include the tumor size, its placement within the HSK, its relationship to the vasculature and the renal collecting system, and the presence of a thick isthmus with a system of straying vessels (20).

3D reconstruction images after helical CT are extremely useful preoperatively as laparoscopic approach commonly used for the treatment of renal carcinoma (1).

## Conclusion

The HSK is the most prevalent congenital urinary tract defect. Because it can be asymptomatic, it is frequently discovered by chance during imaging. The aberrant kidney is typically accompanied by a number of difficulties, including morphological, congenital, and pathologic issues. Particularly, vascular anomalies play a significant role in HSK surgery. A sudden hemorrhage from RCC, a complication which is present in HSK, can endanger the patient’s life during surgery. Because of this, accurate preoperative imaging using current cross-sectional imaging modalities is crucial for preoperative planning.
